# Life Science Research and Drug Discovery at the Turn of the 21st Century: The Experience of SwissBioGrid

**Published:** 2009-04-22

**Authors:** Matthijs den Besten, Arthur J Thomas, Ralph Schroeder

**Affiliations:** 1 Oxford Internet Institute, Oxford University, St Giles, OxfordUnited Kingdom; 2 Oxford -Research Centre, Oxford University, Keble Road, OxfordUnited Kingdom

## Abstract

**Background:**

It is often said that the life sciences are transforming into an information science. As laboratory experiments are starting to yield ever increasing amounts of data and the capacity to deal with those data is catching up, an increasing share of scientific activity is seen to be taking place outside the laboratories, sifting through the data and modelling “in-silico” the processes observed “in-vitro.” The transformation of the life sciences and similar developments in other disciplines have inspired a variety of initiatives around the world to create technical infrastructure to support the new scientific practices that are emerging. The e-Science programme in the United Kingdom and the NSF Office for Cyberinfrastructure are examples of these. In Switzerland there have been no such national initiatives. Yet, this has not prevented scientists from exploring the development of similar types of computing infrastructures. In 2004, a group of researchers in Switzerland established a project, SwissBioGrid, to explore whether Grid computing technologies could be successfully deployed within the life sciences. This paper presents their experiences as a case study of how the life sciences are currently operating as an information science and presents the lessons learned about how existing institutional and technical arrangements facilitate or impede this operation.

**Results:**

SwissBioGrid was established to provide computational support to two pilot projects: one for proteomics data analysis, and the other for high-throughput molecular docking (“virtual screening”) to find new drugs for neglected diseases (specifically, for dengue fever). The proteomics project was an example of a large-scale data management problem, applying many different analysis algorithms to Terabyte-sized datasets from mass spectrometry, involving comparisons with many different reference databases; the virtual screening project was more a purely computational problem, modelling the interactions of millions of small molecules with a limited number of dengue virus protein targets. Both present interesting lessons about how scientific practices are changing when they tackle the problems of large-scale data analysis and data management by means of creating a novel technical infrastructure.

**Conclusions:**

In the experience of SwissBioGrid, data intensive discovery has a lot to gain from close collaboration with industry and harnessing distributed computing power. Yet the diversity in life science research implies only a limited role for generic infrastructure; and the transience of support means that researchers need to integrate their efforts with others if they want to sustain the benefits of their success, which are otherwise lost.

## Background 

The massive amounts of data that science deals with these days have long caught the attention of keen observers. For instance, Chris Anderson posits that the availability of all these data heralds the end of theory [1] and Trefethen and Hey argue that the “data deluge” calls for a new technological infrastructure to support science [2]. Complementing, but not necessarily endorsing these views, in this paper we would like to explore the implications that data driven discovery might have for the organization of the sciences, and in particular the organization of the life sciences.

Whitley has developed a theory that explains the internal structure of different types of sciences on the basis of the level of mutual dependence among scientists and the level of task uncertainty that they face [3]. Dependence can be functional, in the sense that researchers have to use results or procedures from their peers, or strategic, in the sense that researchers have to convince their peers of the value of their research. Task uncertainty in turn can be technical, in the sense that techniques can be more or less well understood and reliable, or strategic, in the sense that problem formulations and significance can be more or less stable. Whitley identifies seven types of social organization to which combinations of task uncertainty and mutual dependence give rise (see Table 1). 

**Table 1:**
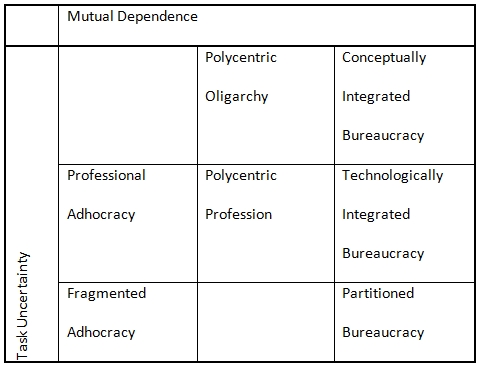
Schematic Representation of Whitley’s (2000) Typology of Social Organization in the Sciences.

For instance, with a low level of uncertainty and a high level of dependence the field of particle physics resembles what Whitley calls a “conceptually integrated bureaucracy” [cf. 4]. At the other end of the spectrum, British sociology, with its focus on diffuse and discursive knowledge of commonsense objects, combines a high level of uncertainty with a low level of dependence in what Whitley calls a “fragmented adhocracy.” According to Wooley and Lin “for much of its history, the organization of biological research could reasonably be regarded as a group of more or less autonomous fiefdoms” [5:p.228; cf. 6: esp.pp.216-40]. This seems to describe what Whitley labels a “polycentric profession.” 

Whitley [3:p. 165] points out that the growth of funding for bio-medical research in the past decades has reduced the degree of strategic dependence among researchers to the extent that the organization of much of the life sciences has now become more like a “professional adhocracy.” Similarly, one might ask what the effect on the social organization of the life sciences might be when we consider the move from observation-lead theorizing to data-driven discovery, which is what the life sciences seem to have gone through in recent years [cf. 7]. “The result of biology’s metamorphosis into an information science just may be the relocation of the lab to the industrial park and the dustbin of history,” writes Timothy Lenoir [7, p. 43]. Yet, if the laboratory is no longer there, it is not clear what replaces it. Table 2 juxtaposes two of the possibilities. 

**Table 2:**
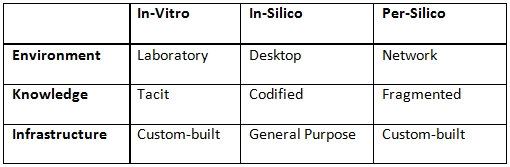
Characteristics of observation- and data-driven discovery.

According to one scenario (in-silico), data-driven discovery takes place in a world where the only resource required is a personal computer; where vast amounts of information are available at one’s fingertips; and where easy to use tools make the manipulation of this information near effortless and amenable to gaining insights on a wide variety of questions. If this were true, the organization of the life sciences would come to resemble a “fragmented adhocracy.” In the other scenario (per-silico), data-driven discovery takes place in a world where all resources are interconnected; where information stems from a myriad of sources; and where considerable efforts go in to the assemblage of tools to manipulate information in a particular way. From this perspective, the only viable organization for the life sciences would seem to be that of a “conceptually integrated bureaucracy” in which large groups try to tackle a small set of clearly defined challenges.

The reality is of course less clear-cut. In their study of the Worm Community System, Star and Ruhleder saw “the emergence of a complex constellation of locally-tailored applications and repositories, combined with pockets of local knowledge and expertise” [8]. And in combination with elements of formal infrastructure this created a “unique and evolving hybrid.” This is what Wooley and Lin may have in mind when they express the hope that “the federation and loose coordination enabled by cyberinfrastructure seem well suited to provide the major advantages of integration while maintaining a reasonably stable large-scale organizational structure” [5: p. 228]. At the same time, even in a world of data-driven discovery, the laboratory does not disappear completely either. As Gardner et al. note: “datasets alone are rarely sufficient to extract and interpret the information provided by the experiment that generated them” [9]. And so, scientists will still need regular contact with the people who carry out real-life experiments in order to interpret their data. 

In order to get a better idea of the social organization engendered by data driven discovery in the life sciences, we need to assess the extent to which laboratories are still required to create and validate scientific knowledge; the extent to which expertise is still required to augment this knowledge; and the extent to which data-discovery infrastructures favour particular directions of research over others. In this paper, we report a case study of a group of researchers in Switzerland who explored how to make most of existing resources and infrastructure in order to carry out data-intensive research. They found that close ties with other professional partners such as industry or specialized research organizations have become important in order to gain access to the relevant data and to ensure that computational results are validated by experimental means. They also found that continued support would be needed if they wanted to allow other researchers to take advantage of the tools that they had developed. Moreover, they found that strategic decisions about which third-party tools to integrate with mattered a lot for the long-term sustainability of their efforts. Thus, while it is hard to say whether task uncertainty and mutual dependence have become larger or smaller on balance for this group of researchers as a result of the move towards data-intensive discovery, it is clear that its character has changed. As our case study of SwissBioGrid will illustrate, it may be difficult to fit attempts to create life science infrastructures for handling data into the models of either an adhocracy or a bureaucracy. Instead, as we shall see, SwissBioGrid had to try to integrate disparate technical elements (software and databases) as well as try to embed these within a more lasting socio-technical structure. It may therefore be necessary to revise Whitley’s scheme to take into account that it is very difficult to integrate research groups within a larger whole in the development of data infrastructures. There are also implications for research policy which is pushing for the development of these infrastructures, an attempt which is likely to encounter continuing tensions between the life sciences as an adhocracy and a bureaucracy. We return to revising Whitley and to this tension in the Discussion and Conclusion. 

## Methods

The material for this case study emerged through participant-observation and interviews, ranging from informal conversations to in-depth interviews. One of the authors of this paper, Thomas, was the SwissBioGrid project coordinator over a 3 year period and therefore in an excellent position as a participant observer. Schroeder and den Besten carried out 3 in depth interviews with the investigators mentioned below.

Key players in the SwissBioGrid project provided us with insights on both the history and background of the project and helped us to try to understand some of the potential impact of SwissBioGrid on computational biology in Switzerland. These included, Torsten Schwede (SIB/Biozentrum): responsible for “prototype 0” of the virtual screening application that had triggered the collaboration; source of more application ideas later in the project; Michael Podvinec (SIB/Biozentrum): molecular biologist; works with Torsten; developed some of the SwissBioGrid infrastructure particularly for using PCs as well as clusters as parts of a computational Grid;Arthur Thomas (CSCS): appointed as project coordinator of SwissBioGrid one year after it started; helped to put it back on track after the project lost some of its early momentum;Peter Kunszt (CSCS): appointed at the same time; in charge of Grid infrastructure.

As part of this case study, we also interviewed several well-known bioinformaticians who were not directly involved in SwissBioGrid, in order to gain a broader perspective on the needs of computational biology. These included Michael Ashburner: professor of genetics at the University of Cambridge; initiator, with others, of the Gene Ontology Consortium, a successful repository of meta-data on genes and their products and reactions; Graham Cameron: a pioneer in the application of database technology in the life sciences and one of the founders of the European Bioinformatics Institute.

The interviews (half an hour with Kunszt on 20.4.2007 by RS; two hours with Schwede and Podvinec on 4.5.2007 by RS and MdB; and two hours with Ashburner and Cameron on 21.5.2007 by AJT, RS, and MdB) were transcribed for analysis and complemented with desk research about the SwissBioGrid, Swiss National e-Research effort and e-Research in the life sciences.

## Results 

In early 2004, Torsten Schwede and Michael Podvinec at the Biozentrum, University of Basel and the Swiss Institute of Bioinformatics (SIB), with encouragement and support from Manuel Peitsch at the Novartis Institute for Biological Research (NIBR), teamed up with Marie-Christine Sawley of the Swiss National Supercomputing Centre (CSCS) in Lugano and formally established a project, which they christened SwissBioGrid [10]. Schwede and Podvinec had been looking for resources and support to carry out virtual screening and Sawley had been thinking about ways to expand the user-base of emerging technologies for Grid computing to the life sciences. So, following the terminology of Hara et al. [11], one could say that both sides were intrinsically motivated to establish this complementary collaboration. Sawley had already helped to establish a high performance computing centre, called Vital-IT, for bioinformatics at SIB in Lausanne, and Schwede and Podvinec has developed close links with Novartis, a major pharmaceutical company, through its Institute of Biomedical Research (NIBR) and the Friedrich Miescher Institute (FMI). When the collaboration was formally established, these institutes as well as the Zurich Functional Genomics Centre (FGCZ), itself a joint venture between the University of Zurich and ETH Zurich, joined in bringing the total number of institutional partners to six. 

At the time, several large-scale funding initiatives were underway or about to get started elsewhere in Europe which aimed to establish national or international Grid-like infrastructures for the sciences [12]. In the United Kingdom, several hundred millions of pounds were being invested in the e-Science Programme, and in the United States efforts were underway by the NSF on a similar scale to develop a cyberinfrastructure. The European Union has since followed suit with funding for e-infrastructure (EGEE). A similar motivation gave rise, four years after the establishment of SwissBioGrid, in 2008, to a new national initiative in Switzerland, the Swiss National Grid Association (SwiNG), funded cooperatively by a number of Swiss universities and aiming to capitalize on the SwissBioGrid and other Swiss initiatives. Around the same time, in 2007, the Swiss National Science Foundation started funding an initiative in systems biology called SystemsX (  http://www.systemsx.ch), in which some of the SwissBioGrid participants also take part. The Swiss National Science Foundation has historically been unwilling to fund infrastructure projects per se [cf. 20]. The fact that the new SystemsX project has explicit, although limited, funding for development of collaborative research platforms may be indicative of a greater appreciation for the needs of collaborative research in Switzerland as well. In early 2009, Kunszt, the SwissBioGrid infrastructure coordinator, became head of the SyBIT IT platform group at SystemsX, giving some continuity of experience from SwissBioGrid into the new organisation.

Between 2004 and 2007, before the Swiss national infrastructure efforts had gotten underway, SwissBioGrid had to rely on local funding and local resources only. There are some negative effects of this, as Podvinec pointed out:

This grassroots way of operating has also had a lot of problems. People are going to be motivated by their own interests foremost, and only contribute when you really force them to. This will lead to broken promises, to delays and to parts of the common infrastructure that never get properly done.

But this did not stall developments. In early 2006 Arthur Thomas (one of the authors of this case study) became the Project Coordinator, and soon thereafter the collaboration was expanded to include the Proteome Informatics Group of the SIBat the University of Geneva, and the ETH Zurich Institute of Molecular Systems Biology, who were also looking for computing resources large enough to tackle the enormous (Terabyte-scale) amounts of data beginning to emerge from high-throughput proteomics studies, and which needed to be analysed in many different ways, involving parallel application of different algorithms. GeneBio SA, a Geneva-based company, which produces a widely-used commercial proteomics analysis suite, Phenyx, provided substantial technical support and favourable licensing terms to make Phenyx accessible to SwissBioGrid. Schrödinger LLC, a commercial company which develops the widely-used Glide package for modelling ligand drug/protein binding, also cooperated to make their software available on favourable licensing terms. And so SwissBioGrid served as a sand-box in which a multitude of players could experiment with new technologies for distributed computing. Scientifically, the collaboration paid off as well as a combined effort of about 2 dedicated full-time- equivalent researchers per year resulted in a grand total of 32 publications in the three years that SwissBioGrid operated.

### The SwissBioGrid Research Environment

From a technical perspective, the main goal of SwissBioGrid was to develop an infrastructure that could use the distributed computing capacity of clusters and PCs at the partner institutions to solve hard computational problems in biology. This capacity included Unix clusters at Vital-IT, CSCS, the Biozentrum, the University of Zurich and the ETH Zurich, and PC farms at the University of Basel, NIBR and FMI. Scientifically, this infrastructure served two goals. First it served as an environment for high-throughput molecular docking (“virtual screening”), to find new drugs for neglected diseases (specifically, for dengue fever, which is widespread in mosquito-ridden areas of the world) and later it served as environment for proteomics data analysis as well. The proteomics project was an example of a data management problem, applying many different analysis algorithms to Terabyte-sized datasets from mass spectrometry, involving comparisons with many different reference databases; the virtual screening project was more a purely computational problem, modelling the interactions of millions of small molecules with a limited number of protein targets on the coat of the dengue virus.

Yet, it would be wrong to equate the research environment with the computing environment. Crucial for the scientific success of the virtual screening project was that Novartis, the partner from the pharmaceutical industry, further agreed that its Tropical Diseases Research Institute in Singapore would perform experimental studies on any leads that emerged from the virtual screening experiments. Novartis further committed itself to provide any drugs which resulted from this collaboration at cost to dengue sufferers in the developing world. This project was thus a good example of a public-private partnership for the public good. In the experience of Schwede and Podvinec, many bioinformatics studies deliver DVDs full of data that are never looked at. As Schwede puts it:

There have been a number of projects that have done, or have tried to do computational drug discovery… but which end up with saying “We have been very successful; we produced 30 DVDs full of results.” And that’s as far as it goes. It usually has a footnote: “these are now very valuable results for biologists who will test this.”… or not.

The collaboration with Novartis made sure that the results discovered on the computer were validated in reality. In return, Novartis was given the exclusive right to exploit the results. Because the dengue virus was of limited commercial interest to Novartis, establishing a public-private partnership with them was relatively easy. Even so, the legal negotiations for this part of the project were the most difficult agreements in the whole project.

### SwissBioGrid Expertise

In the course of the project, significant software development efforts were undertaken by the Biozentrum, where Podvinec developed a mechanism for dispatching jobs to a mixed grid of desktop PCs and Linux clusters, and by CSCS, who managed the overall Grid infrastructure. For some key technologies the best solutions were only available from commercial vendors. Unfortunately, most existing licensing models turned out to be incompatible with Grid computing since they relied on technical assumptions not necessarily satisfied in a Grid environment (such as continuous network connectivity to a license server), or on pricing schemes originating from the pre-Grid era (e.g. per-CPU licenses). But luckily, at least the two companies already mentioned, GeneBio and Schödinger, were prepared to provide their software on favourable terms. More cumbersome than the licenses however, were the differences among software and database interfaces that had to be bridged, the numerical stability of software in heterogeneous environments, and the problem of data caching.

The incompatibility of databases is the result of how they came into being: there are many small groups working on separate databases, which use different language to describe their content (or even in some cases the same language to describe different content!). Similarly, software in computational biology and bioinformatics has often been developed on a single computational platform without much emphasis on portability or scalability. Many software packages used in this field give different numerical results when executed on different hardware or software platforms. These differences are often not negligible, and might lead to contradictory scientific conclusions depending on the platform where a calculation was performed. And so, validation of numerical stability turned out to be essential for using heterogeneous Grid architectures. A final obstacle was the need to ensure that up-to-date, synchronized copies of the needed databases are available “on demand” at the computational nodes. Their large size precludes either keeping copies everywhere, or moving copies to the computational nodes “just in time”. For the time being, the problem of optimizing database distribution (sometimes called the “data caching” problem) remains without a satisfactory generic solution. And so, SwissBioGrid had to develop specific solutions to that problem as well. 

 From the perspective of Schwede and Podvinec in particular, the technical expertise developed through SwissBioGrid represented a somewhat wasted effort in two ways. First of all, they were unlikely to receive much scientific recognition for all the efforts that went into making the tools work before they started to produce results. This is a typical problem in e-Science or cyberinfrastructure, where there is little reward for tool development as opposed to high-ranking publications within the researcher’s own discipline. Secondly, the extent of adaptations that needed to be made and the brittleness of the system that resulted meant that their continued expertise would be required if the wanted to make their tools work for other users or in other environments. It could be worse, Schwede acknowledged:

At this stage we are very lucky because it is kind of pioneering work. But let’s imagine we would have been successful in providing a service structure: running an infrastructure service is much more work than building a prototype. And if you run a service that is acting in the background - running as middleware - you don’t get any recognition for this at all.

Yet, the motivation for Schwede and Podvinec to engage with the Grid in the first place was the hope that it would provide a more user-friendly environment that others would then maintain.

### SwissBioGrid Infrastructure

At the start of the project, SwissBioGrid hoped to take advantage of “middleware”, i.e., system software for the management of distributed resources, that was being developed under the auspices of the European Union sponsored EGEE project for the analysis of observations from CERN’s Large Hadron Collider [12]. However, in the event the NorduGrid/ARC suite was chosen as middleware, since the more widely-used EGEE /gLite suite appeared, after a comprehensive evaluation performed by CSCS and Vital-IT, to be too complex, inflexible and intrusive, especially when PCs are also used as computing resources. In addition, since biological problems tend to involve more complex and heterogeneous data than many in the physical sciences for which both NorduGrid/Arc and EGEE/gLite had been developed, the project had to overcome substantial challenges in distributed data management such as the “data-caching” problem mentioned before, before being able to deploy the middleware. As Podvinec explains:

I think that the reason why we started with the dengue project is because it is so close to High Energy Physics that in the end you can see it as another incarnation of this needle-in-the haystack problem with just a little bit of bio-typical problematic in it […] But if you take the next step, as in the proteomics project, you already need the context of all known genomes for a search, and you need to distribute these large datasets to all computers running the computation.

In this context, NorduGrid/Arc had the additional benefit that its developer community was relatively small and hence more open to demands for features that were necessary to make it work for SwissBioGrid. Yet, in the long run this benefit proved to be a disadvantage as uncertainty about funding for continued development of NorduGrid/Arc made it unlikely that the tools that were so well integrated with this suite would remain easy to deploy in future.

## Discussion 

Data driven discovery, in the experience of SwissBioGrid, is a collaborative effort, which includes many partners from industry as well as academia. In fact, the scale and capital requirements of modern laboratory research make ties with industry almost indispensable. Much of the expertise that is accumulated in the process of discovery relates to tool development. As such, the scientific value of this expertise is not always immediately obvious, as it relates to matters that are often at best peripheral to the concerns of biologists. And this, in turn, makes it hard to obtain credit for tool development, as opposed to the scientific results that are subsequently derived. Moreover, tool development is not limited to the outer layer of end-user applications. SwissBioGrid found that it is not always safe to assume that software infrastructure for distributed computing that has been developed for data driven discovery in, say, particle physics, can be adopted without substantial adjustments in the context of the life sciences. The heterogeneity of resources in the life sciences and the recurring need to combine multiple resources are major factors that make the establishment of a single infrastructure difficult. As Cameron explains:

The behaviour of the molecules themselves is very complicated and the kind of information that we can collect on it is very complicated; there are actually a lots of bits of biology that sort of talk to each other – there are always the DNA sequencing guys; there are the protein guys; there are the molecular interaction guys; there are the pathway guys – they’re all talking about a common set of entities in a living system; and they are collecting what is currently rather patchy information, scientific information, about those; and you want, both within any individual collection, if you’re talking about a given protein product, you want to call it the same thing, the connections between all those entities come from the identifiers and the knowledge attached. […] There are a lot of places where it is relatively easy, [others where] you’d discover that biology did something that you didn’t anticipate. So, the structure is constantly evolving; the entities that are in there are connected to each other; you have to share the data-structures, and you have to share the way you refer to the entities. And the entities don’t stand still either. You know, it is commonplace for people to be blathering on about some gene or other and a couple of variants of it only to discover that actually it wasn’t one gene that they were talking about, but two different genes.

The life sciences make use of a wide variety of databases (using the term in a rather loose, non-technical sense) in a wide variety of formats and, more importantly, with a wide range of semantics. So integrating information from different databases is made more difficult by the difficulties of reconciling those semantic differences. New tools, largely coming from the “ontological engineering” community, hold out the promise of being at the very least able to identify inconsistencies of nomenclature between different databases, and perhaps even helping to reconcile those differences. Significant effort (exemplified by the Open Biological Ontologies project- http://www.obofoundry.org) is now being put into addressing this problem. As Cameron puts it:

We need to connect the databases with each other so that … not only can people find things of interest but they can build tools, which are satisfactory in terms of the visualization of that information.

In SwissBioGrid, the proteomics project in particular was challenged by the need to compare the experimental data with reference (peptide fragment) data from a number of different sources, and also with merging the results of different analysis methods to give a coherent and integrated view of the results. 

Moreover, the problem of establishing appropriate reward structures is unsolved. Many academic institutions are wrestling with this problem internally. New, but as yet very immature, developments in Wiki-based collaborative knowledge creation (such as WikiGenes, WikiProteins) make possible the explicit tracking and visualization of individual contributions to the knowledge repositories; how this information can be applied to traditional methods of impact analysis remains to be seen.

Consequently, the organizational structure that typifies research in SwissBioGrid is difficult to pin down as well. The laboratory no longer seems to occupy the dominant place that it occupied several decades ago when the life sciences could be characterized as a “polycentric profession.” Yet, reduced dependence vis-à-vis the principal investigator of a single laboratory (as described in [6]) seems to have been replaced by new dependencies due to the need to retain access to data, ensure the validation of results, and the need to sustain research procedures in changing environments. So one could not typify this research organization as an “adhocracy” either. Nor does it look like a “conceptually integrated bureaucracy,” as the life sciences lack the clarity of goals and a dominant agenda-setting research institute that sets those goals. Perhaps Whitley’s own classification of the life sciences as a “polycentric profession” still applies. Alternatively, if the visions of cyberinfrastructure, e-science and, more recently, SwiNG materialise, the life sciences would be part of nationally and internationally organized technologically integrated bureaucracies. Yet, if anything, the experience of SwissBioGrid highlights the difficulty of technological integration without conceptual integration.

Hence, from a research policy perspective one might wonder whether it would not be better for the life sciences if their research organization could be transformed into the conceptually integrated bureaucracy that is more typical of particle physics or into the fragmented adhocracy that is found in sociology: a bureaucracy might be more efficient (and thus allow for the creation of a central information or data infrastructure). Yet an adhocracy might be more diverse (and thus require a more flexible infrastructure or simple federated resources). The life sciences could become more like a conceptually integrated bureaucracy if relatively big institutions, such as the US National Center for Biotechnology Information, the European Bioinformatics Institute or the Wellcome Trust Sanger Institute, were able to secure more substantial long term funding to tackle infrastructure issues, as opposed to purely scientific ones. They would then attract the necessary support staff to develop the custom infrastructures needed to carry out this research, much like CERN in the context of particle physics. Alternatively, the life sciences could become more like an adhocracy if more efforts were made to guarantee easy and equal access to data, publications, and tools. The newer paradigm of “cloud computing” might provide computing to researchers-at-large where the Grid paradigm appears to have fallen short, or the wrapping of research tools and data as web services might make new configurations for research easier to achieve. In addition, substantial investment in traditional laboratory facilities would be required to promote the “in-vitro” validation of interesting “in-silico” research. At least part of these facilities should be non-commercial, as not everything that is interesting necessarily yields a profit.

But perhaps it is not necessary or even possible to push the life sciences into the mould of an adhocracy or a bureaucracy. Vallas and Kleinman [13] characterize the life sciences as a “heterarchy” – an increasingly interconnected scientific field in which the distinctions between academic and corporate sciences are blurred and which is marked by the anomalies, tensions and ironies that were already observed by Hackett [14]. For research that is so clearly located in what Stokes [15] called Pasteur’s quadrant of use-inspired basic research as opposed to “pure” basic research, it is hard to see how one could exclude corporate interests, even if one wanted to. The question is rather, if the academic and corporate spheres are no longer separate, what organizational principles should govern the common sphere that emerges, and how should potentially contentious issues like intellectual property ownership be settled? Vallas and Kleinman argue that the convergence to date has been asymmetrical in that universities were more eager to adopt the behaviour of industry than vice-versa [13]. There may however be reasons for hope that industry will increasingly adopt an academic style of research as well focusing on open innovation in basic research such that it can reap more benefits from the results [16]. 

## Conclusions 

In this paper, we have characterized the organization of the life sciences to which data driven discovery can give rise using the typology proposed by Whitley [3] and looked at the organization of research in one particular project, SwissBioGrid. What we found is a project that was the product of a collaboration that spanned multiple places and disciplines and relied on the synergy of corporate and academic research. It also required a level of technical integration that is difficult to achieve. 

Recently, in the life sciences and elsewhere, there have been attempts at ‘community’ based (for example [19]) and open-source [16] alternatives to the challenge of creating infrastructures for data management and data analysis, but these are still in very early phases and it remains to be seen if they will be successful in addressing these challenges.

In our case, it is worth mentioning the public-private partnership between the Biozentrum, Schrödinger LLC and the Novartis Institute for Tropical Diseases. that was crucial to SwissBioGrid in terms of distributed computing. This type of industry-university interaction is often seen as key challenge in how research is changing (it has been explored, for example, by Forte, Lenoir, and their colleagues [17,18]) and may be important elsewhere. Yet we found that this interaction did not present a particular problem in the case of SwissBioGrid. The challenge came instead from the technical and organizational issues of data management and computational problems in the life sciences. We highlighted the successes in tackling these, but also the heterogeneity of the life sciences and the lack of an infrastructure in which the solutions developed in SwissBioGrid could be sustained. 
